# 2-[(4-Chloro­benzyl­idene)amino]-4,5,6,7-tetra­hydro-1-benzothio­phene-3-carbonitrile

**DOI:** 10.1107/S1600536811030704

**Published:** 2011-08-06

**Authors:** Abdullah M. Asiri, Salman A. Khan, M. Nawaz Tahir

**Affiliations:** a The Center of Excellence for Advanced Materials Research, King Abdulaziz University, Jeddah 21589, PO Box 80203, Saudi Arabia; b Department of Chemistry, Faculty of Science, King Abduaziz University, Jeddah 21589, PO Box 80203, Saudi Arabia; c University of Sargodha, Department of Physics, Sargodha, Pakistan

## Abstract

In the title compound, C_16_H_13_ClN_2_S, the dihedral angle between the 4-chloro­benzaldehyde moiety and the heterocyclic five-membered ring is 7.21 (17)°. In the crystal, mol­ecules are linked by weak C—H⋯π inter­actions, generating [100] chains.

## Related literature

For a related structure, see: Asiri *et al.* (2011 ▶).
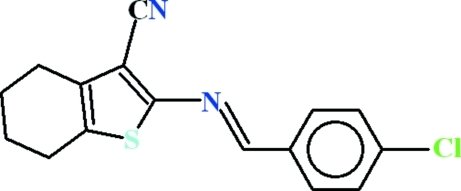


## Experimental

### 

#### Crystal data

C_16_H_13_ClN_2_S
*M* *_r_* = 300.79
Orthorhombic, 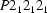

*a* = 4.7815 (3) Å
*b* = 16.5670 (13) Å
*c* = 18.1658 (14) Å
*V* = 1439.01 (18) Å^3^
*Z* = 4
Mo *K*α radiation
μ = 0.40 mm^−1^
*T* = 296 K
0.35 × 0.15 × 0.12 mm

#### Data collection

Bruker Kappa APEXII CCD diffractometer
Absorption correction: multi-scan (*SADABS*; Bruker, 2005 ▶) *T*_min_ = 0.931, *T*_max_ = 0.951
11075 measured reflections
2607 independent reflections
1821 reflections with *I* > 2σ(*I*)
*R*_int_ = 0.055

#### Refinement

*R*[*F* ^2^ > 2σ(*F* ^2^)] = 0.044
*wR*(*F* ^2^) = 0.099
*S* = 1.02
2607 reflections
181 parameters
H-atom parameters constrained
Δρ_max_ = 0.26 e Å^−3^
Δρ_min_ = −0.19 e Å^−3^
Absolute structure: Flack (1983 ▶), 1053 Friedel pairs
Flack parameter: 0.03 (10)

### 

Data collection: *APEX2* (Bruker, 2009 ▶); cell refinement: *SAINT* (Bruker, 2009 ▶); data reduction: *SAINT*; program(s) used to solve structure: *SHELXS97* (Sheldrick, 2008 ▶); program(s) used to refine structure: *SHELXL97* (Sheldrick, 2008 ▶); molecular graphics: *ORTEP-3 for Windows* (Farrugia, 1997 ▶) and *PLATON* (Spek, 2009 ▶); software used to prepare material for publication: *WinGX* (Farrugia, 1999 ▶) and *PLATON*.

## Supplementary Material

Crystal structure: contains datablock(s) global, I. DOI: 10.1107/S1600536811030704/hb6331sup1.cif

Structure factors: contains datablock(s) I. DOI: 10.1107/S1600536811030704/hb6331Isup2.hkl

Supplementary material file. DOI: 10.1107/S1600536811030704/hb6331Isup3.cml

Additional supplementary materials:  crystallographic information; 3D view; checkCIF report

## Figures and Tables

**Table 1:** Hydrogen-bond geometry (Å, °) *Cg* is the centroid of the C8–C11/S1 ring.

*D*—H⋯*A*	*D*—H	H⋯*A*	*D*⋯*A*	*D*—H⋯*A*
C13—H13*A*⋯*Cg*^i^	0.97	2.99	3.841 (6)	147

Symmetry code: (i) 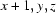.

**Fig. 1.**
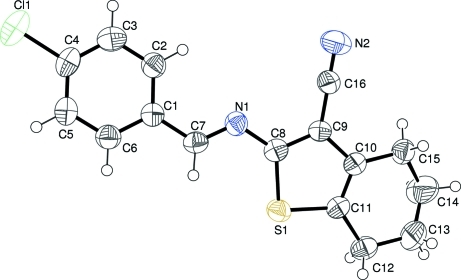
View of (I) showing 50% displacement ellipsoids.

## supplementary crystallographic information

### Comment

We have reported the crystal structure of 2-[(benzo[1,3]dioxol-5-ylmethylene)-amino]-4,5,6,7-tetrahydro-benzo[*b*]thiophene -3-carbonitrile (Asiri *et al.*, 2011) which is related to the title compound, (I), Fig. 1.

In (I), the group A (C1–C7/CL1) of 4-chlorobenzaldehyde and the five membered ring B (C8—C11/S1) of 2-amino-4,5,6,7-tetrahydro-1-benzothiophene-3- carbonitrile group are almost planar with r. m. s. deviation of 0.0150 and 0.0110 Å, respectively. The dihedral angle between A/B is 7.21 (17)°. A C—H···π interaction (Table 1) occurs in the crystal.

### Experimental

A mixture of 4-chloro benzaldehyde (0.46 g, 2.4 mmol) and 2-amino-4,5,6,7-tetrahydro-benzo[*b*]thiophene-carbonitrile (0.32 g, 3.3 mmol) in ethanol (15 ml) was heated for 3 h. The progress of the reaction was monitored by TLC. The solid that separated from the cooled mixture was collected and recrystallized from a methanol-chloroform mixture (8:2) to give yellow needles of the title compound (I). Yield: 82%, m.p. 504–505 K.

### Refinement

The H-atoms were positioned geometrically (C–H = 0.93–0.97 Å) and refined as riding with *U*_iso_(H) = 1.2*U*_eq_(C).

### Crystal data

**Table:**

C_16_H_13_ClN_2_S	*F*(000) = 624
*M**_r_* = 300.79	*D*_x_ = 1.388 Mg m^−^^3^
Orthorhombic, *P*2_1_2_1_2_1_	Mo *K*α radiation, λ = 0.71073 Å
Hall symbol: P 2ac 2ab	Cell parameters from 1821 reflections
*a* = 4.7815 (3) Å	θ = 3.3–25.2°
*b* = 16.5670 (13) Å	µ = 0.40 mm^−^^1^
*c* = 18.1658 (14) Å	*T* = 296 K
*V* = 1439.01 (18) Å^3^	Needle, yellow
*Z* = 4	0.35 × 0.15 × 0.12 mm

### Data collection

**Table:**

Bruker Kappa APEXII CCD diffractometer	2607 independent reflections
Radiation source: fine-focus sealed tube	1821 reflections with *I* > 2σ(*I*)
graphite	*R*_int_ = 0.055
Detector resolution: 8.20 pixels mm^-1^	θ_max_ = 25.2°, θ_min_ = 3.3°
ω scans	*h* = −5→5
Absorption correction: multi-scan (*SADABS*; Bruker, 2005)	*k* = −19→17
*T*_min_ = 0.931, *T*_max_ = 0.951	*l* = −21→21
11075 measured reflections	

### Refinement

**Table:**

Refinement on *F*^2^	Secondary atom site location: difference Fourier map
Least-squares matrix: full	Hydrogen site location: inferred from neighbouring sites
*R*[*F*^2^ > 2σ(*F*^2^)] = 0.044	H-atom parameters constrained
*wR*(*F*^2^) = 0.099	*w* = 1/[σ^2^(*F*_o_^2^) + (0.0416*P*)^2^ + 0.1311*P*] where *P* = (*F*_o_^2^ + 2*F*_c_^2^)/3
*S* = 1.02	(Δ/σ)_max_ < 0.001
2607 reflections	Δρ_max_ = 0.26 e Å^−^^3^
181 parameters	Δρ_min_ = −0.19 e Å^−^^3^
0 restraints	Absolute structure: Flack (1983), 1053 Friedel pairs
Primary atom site location: structure-invariant direct methods	Flack parameter: 0.03 (10)

### Special details

**Table:**

Geometry. Bond distances, angles *etc*. have been calculated using the rounded fractional coordinates. All su's are estimated from the variances of the (full) variance-covariance matrix. The cell e.s.d.'s are taken into account in the estimation of distances, angles and torsion angles
Refinement. Refinement of *F*^2^ against ALL reflections. The weighted *R*-factor *wR* and goodness of fit *S* are based on *F*^2^, conventional *R*-factors *R* are based on *F*, with *F* set to zero for negative *F*^2^. The threshold expression of *F*^2^ > σ(*F*^2^) is used only for calculating *R*-factors(gt) *etc*. and is not relevant to the choice of reflections for refinement. *R*-factors based on *F*^2^ are statistically about twice as large as those based on *F*, and *R*- factors based on ALL data will be even larger.

### Fractional atomic coordinates and isotropic or equivalent isotropic displacement parameters (Å^2^)

**Table:**

	*x*	*y*	*z*	*U*_iso_*/*U*_eq_	
Cl1	−0.7805 (2)	−0.17259 (7)	−0.04726 (7)	0.0797 (4)	
S1	0.4743 (2)	0.20961 (5)	0.08062 (4)	0.0506 (3)	
N1	0.1842 (6)	0.06647 (16)	0.10865 (15)	0.0453 (10)	
N2	0.4578 (9)	0.0048 (2)	0.28643 (17)	0.0787 (14)	
C1	−0.1473 (7)	0.0110 (2)	0.02527 (17)	0.0406 (11)	
C2	−0.2345 (8)	−0.0493 (2)	0.07260 (19)	0.0503 (12)	
C3	−0.4313 (7)	−0.1048 (2)	0.0506 (2)	0.0547 (12)	
C4	−0.5384 (7)	−0.1013 (2)	−0.01982 (19)	0.0513 (12)	
C5	−0.4597 (8)	−0.0416 (2)	−0.06685 (19)	0.0560 (12)	
C6	−0.2640 (7)	0.0135 (2)	−0.04455 (18)	0.0510 (12)	
C7	0.0619 (7)	0.06949 (19)	0.04718 (19)	0.0445 (11)	
C8	0.3759 (7)	0.1241 (2)	0.12951 (18)	0.0443 (12)	
C9	0.5135 (8)	0.12209 (18)	0.19578 (15)	0.0393 (10)	
C10	0.6876 (6)	0.19068 (19)	0.20924 (17)	0.0403 (11)	
C11	0.6880 (7)	0.24227 (19)	0.15209 (17)	0.0417 (12)	
C12	0.8316 (8)	0.3222 (2)	0.15026 (19)	0.0540 (12)	
C13	1.0043 (12)	0.3330 (3)	0.2182 (3)	0.0947 (19)	
C14	0.9144 (12)	0.2949 (3)	0.2830 (2)	0.103 (2)	
C15	0.8375 (7)	0.2073 (2)	0.27953 (18)	0.0527 (12)	
C16	0.4818 (9)	0.0573 (2)	0.24609 (17)	0.0484 (11)	
H2	−0.15934	−0.05237	0.11974	0.0602*	
H3	−0.49192	−0.14449	0.08309	0.0652*	
H5	−0.53781	−0.03830	−0.11363	0.0670*	
H6	−0.20794	0.05376	−0.07708	0.0609*	
H7	0.10771	0.11083	0.01472	0.0534*	
H12A	0.69350	0.36500	0.14716	0.0649*	
H12B	0.95053	0.32546	0.10711	0.0649*	
H13A	1.19183	0.31404	0.20749	0.1138*	
H13B	1.01751	0.39043	0.22811	0.1138*	
H14A	0.75291	0.32421	0.30118	0.1240*	
H14B	1.06135	0.30081	0.31940	0.1240*	
H15A	1.00517	0.17449	0.28231	0.0631*	
H15B	0.71853	0.19360	0.32092	0.0631*	

### Atomic displacement parameters (Å^2^)

**Table:**

	*U*^11^	*U*^22^	*U*^33^	*U*^12^	*U*^13^	*U*^23^
Cl1	0.0610 (7)	0.0690 (7)	0.1092 (9)	−0.0156 (6)	−0.0022 (6)	−0.0261 (6)
S1	0.0612 (6)	0.0437 (5)	0.0469 (4)	−0.0018 (5)	−0.0048 (5)	0.0056 (4)
N1	0.0506 (18)	0.0409 (18)	0.0444 (16)	0.0006 (16)	−0.0036 (14)	−0.0047 (13)
N2	0.105 (3)	0.069 (2)	0.062 (2)	−0.014 (2)	−0.007 (2)	0.0196 (19)
C1	0.0409 (19)	0.036 (2)	0.045 (2)	0.0070 (17)	0.0009 (15)	−0.0040 (16)
C2	0.057 (2)	0.046 (2)	0.048 (2)	0.0009 (19)	−0.0022 (19)	−0.0042 (18)
C3	0.058 (2)	0.044 (2)	0.062 (2)	0.005 (2)	0.011 (2)	0.0012 (19)
C4	0.040 (2)	0.046 (2)	0.068 (2)	0.002 (2)	0.001 (2)	−0.0177 (19)
C5	0.054 (2)	0.060 (2)	0.054 (2)	−0.004 (2)	−0.009 (2)	−0.0066 (19)
C6	0.056 (2)	0.048 (2)	0.049 (2)	−0.001 (2)	0.001 (2)	0.0010 (18)
C7	0.050 (2)	0.038 (2)	0.0454 (18)	−0.0005 (18)	0.0050 (19)	0.0018 (16)
C8	0.046 (2)	0.040 (2)	0.047 (2)	0.0011 (18)	0.0043 (16)	−0.0030 (16)
C9	0.0423 (19)	0.0362 (18)	0.0394 (17)	0.0020 (18)	0.0040 (17)	0.0007 (14)
C10	0.0350 (18)	0.040 (2)	0.0459 (19)	0.0040 (18)	0.0049 (15)	−0.0024 (17)
C11	0.040 (2)	0.036 (2)	0.049 (2)	0.0008 (17)	0.0045 (16)	−0.0023 (17)
C12	0.056 (2)	0.042 (2)	0.064 (2)	−0.001 (2)	0.0060 (19)	0.0011 (18)
C13	0.112 (4)	0.070 (3)	0.102 (3)	−0.044 (3)	−0.038 (4)	0.007 (3)
C14	0.154 (5)	0.092 (4)	0.064 (3)	−0.063 (4)	−0.007 (3)	−0.012 (3)
C15	0.051 (2)	0.056 (2)	0.051 (2)	−0.005 (2)	−0.0019 (17)	−0.0042 (19)
C16	0.055 (2)	0.050 (2)	0.0403 (18)	−0.005 (2)	−0.0030 (19)	−0.0001 (17)

### Geometric parameters (Å, °)

**Table:**

Cl1—C4	1.727 (4)	C11—C12	1.492 (5)
S1—C8	1.737 (3)	C12—C13	1.496 (7)
S1—C11	1.739 (3)	C13—C14	1.403 (7)
N1—C7	1.262 (4)	C14—C15	1.499 (6)
N1—C8	1.377 (4)	C2—H2	0.9300
N2—C16	1.143 (5)	C3—H3	0.9300
C1—C2	1.382 (5)	C5—H5	0.9300
C1—C6	1.386 (5)	C6—H6	0.9300
C1—C7	1.448 (5)	C7—H7	0.9300
C2—C3	1.375 (5)	C12—H12A	0.9700
C3—C4	1.379 (5)	C12—H12B	0.9700
C4—C5	1.360 (5)	C13—H13A	0.9700
C5—C6	1.369 (5)	C13—H13B	0.9700
C8—C9	1.372 (4)	C14—H14A	0.9700
C9—C10	1.430 (4)	C14—H14B	0.9700
C9—C16	1.418 (4)	C15—H15A	0.9700
C10—C11	1.345 (4)	C15—H15B	0.9700
C10—C15	1.490 (4)		
			
C8—S1—C11	91.79 (15)	C1—C2—H2	120.00
C7—N1—C8	121.7 (3)	C3—C2—H2	120.00
C2—C1—C6	118.0 (3)	C2—C3—H3	120.00
C2—C1—C7	121.4 (3)	C4—C3—H3	120.00
C6—C1—C7	120.6 (3)	C4—C5—H5	120.00
C1—C2—C3	120.6 (3)	C6—C5—H5	120.00
C2—C3—C4	119.7 (3)	C1—C6—H6	119.00
Cl1—C4—C3	119.2 (3)	C5—C6—H6	119.00
Cl1—C4—C5	120.1 (3)	N1—C7—H7	119.00
C3—C4—C5	120.7 (3)	C1—C7—H7	119.00
C4—C5—C6	119.2 (3)	C11—C12—H12A	110.00
C1—C6—C5	121.7 (3)	C11—C12—H12B	110.00
N1—C7—C1	122.5 (3)	C13—C12—H12A	110.00
S1—C8—N1	127.3 (2)	C13—C12—H12B	110.00
S1—C8—C9	109.8 (2)	H12A—C12—H12B	108.00
N1—C8—C9	123.0 (3)	C12—C13—H13A	108.00
C8—C9—C10	114.2 (3)	C12—C13—H13B	108.00
C8—C9—C16	122.2 (3)	C14—C13—H13A	108.00
C10—C9—C16	123.6 (3)	C14—C13—H13B	108.00
C9—C10—C11	112.0 (3)	H13A—C13—H13B	107.00
C9—C10—C15	125.0 (3)	C13—C14—H14A	108.00
C11—C10—C15	122.9 (3)	C13—C14—H14B	108.00
S1—C11—C10	112.2 (2)	C15—C14—H14A	108.00
S1—C11—C12	122.0 (2)	C15—C14—H14B	108.00
C10—C11—C12	125.6 (3)	H14A—C14—H14B	107.00
C11—C12—C13	110.0 (3)	C10—C15—H15A	110.00
C12—C13—C14	118.0 (5)	C10—C15—H15B	110.00
C13—C14—C15	118.4 (4)	C14—C15—H15A	110.00
C10—C15—C14	109.5 (3)	C14—C15—H15B	110.00
N2—C16—C9	179.5 (4)	H15A—C15—H15B	108.00
			
C11—S1—C8—N1	176.1 (3)	S1—C8—C9—C10	2.8 (4)
C11—S1—C8—C9	−2.1 (3)	S1—C8—C9—C16	−177.6 (3)
C8—S1—C11—C10	0.8 (3)	N1—C8—C9—C10	−175.4 (3)
C8—S1—C11—C12	−174.3 (3)	N1—C8—C9—C16	4.2 (5)
C8—N1—C7—C1	−177.9 (3)	C8—C9—C10—C11	−2.3 (4)
C7—N1—C8—S1	2.5 (5)	C8—C9—C10—C15	173.8 (3)
C7—N1—C8—C9	−179.6 (3)	C16—C9—C10—C11	178.1 (3)
C6—C1—C2—C3	−0.1 (5)	C16—C9—C10—C15	−5.8 (5)
C7—C1—C2—C3	−179.3 (3)	C9—C10—C11—S1	0.6 (4)
C2—C1—C6—C5	0.0 (5)	C9—C10—C11—C12	175.6 (3)
C7—C1—C6—C5	179.3 (3)	C15—C10—C11—S1	−175.6 (2)
C2—C1—C7—N1	3.4 (5)	C15—C10—C11—C12	−0.6 (5)
C6—C1—C7—N1	−175.9 (3)	C9—C10—C15—C14	−160.7 (3)
C1—C2—C3—C4	1.4 (5)	C11—C10—C15—C14	15.0 (5)
C2—C3—C4—Cl1	178.8 (3)	S1—C11—C12—C13	−178.9 (3)
C2—C3—C4—C5	−2.6 (5)	C10—C11—C12—C13	6.6 (5)
Cl1—C4—C5—C6	−178.9 (3)	C11—C12—C13—C14	−29.9 (6)
C3—C4—C5—C6	2.5 (5)	C12—C13—C14—C15	49.0 (7)
C4—C5—C6—C1	−1.2 (5)	C13—C14—C15—C10	−38.8 (6)

### Hydrogen-bond geometry (Å, °)

**Table:**

Cg is the centroid of the C8–C11/S1 ring.

**Table:**

*D*—H···*A*	*D*—H	H···*A*	*D*···*A*	*D*—H···*A*
C13—H13A···Cg^i^	0.97	2.99	3.841 (6)	147

Symmetry codes: (i) *x*+1, *y*, *z*.
